# College students’ interpretations of food security questions: results from cognitive interviews

**DOI:** 10.1186/s12889-019-7629-9

**Published:** 2019-10-11

**Authors:** Cassandra J. Nikolaus, Brenna Ellison, Sharon M. Nickols-Richardson

**Affiliations:** 1 Institute for Research and Education to Advance Community Health, Washington State University, 1100 Olive Way, Suite 1200, Seattle, WA 98101 USA; 2 Department of Agricultural and Consumer Economics, University of Illinois at Urbana-Champaign, 1301 W. Gregory Dr, Urbana, IL 61801 USA; 3 University of Illinois Extension & Outreach, University of Illinois at Urbana-Champaign, 1301 W. Gregory Dr, Urbana, IL 61801 USA

**Keywords:** Food insecurity, Cognitive interviews, Qualitative research, University students

## Abstract

**Background:**

Food insecurity (FI) – the lack of sufficient access to food to maintain a healthy lifestyle – among college (i.e. post-secondary or higher education institution) students has become a prominent issue in the U.S. However, it is not clear if high rates of FI among students are due to the modern experience in higher education institutions or due to underlying issues in common surveying methods. To understand if there were underlying content validity issues, the present study had two primary research questions: 1) How do students interpret the U.S. Department of Agriculture (USDA) Food Security Survey Module (FSSM) questionnaire items, and 2) How do responses of students experiencing FI compare with the theorized experiences and coping responses?

**Methods:**

Thirty-three undergraduate students, aged 18- to 24-years old and fluent in English were recruited from a single 4-year university. During a 60-min session, participants completed the 10-item Adult FSSM and then were cognitively interviewed about their responses using the think-aloud method. Interview transcripts were analysed by two researchers using a collaborative process and basic interpretative approach.

**Results:**

Students were on average 19.5 years old (± 1.2 years), the majority were in their freshman or sophomore (i.e., first or second) year, and 67% (*n* = 22) experienced FI. Results indicated that students’ interpretations of key terms – such as “money for more,” “balanced meals,” and “real hunger” – diverge from expectations. Furthermore, students categorized as food insecure reported experiences and responses to FI that varied from theoretical dimensions of the process.

**Conclusions:**

Though limited by sample size and representativeness, the present results indicate that the content validity of the FSSM may be compromised in this population and the managed process of FI may present differently among college students. Further psychometric research on modifications to the FSSM or with new FI assessment tools should be conducted with college students.

## Background

Food insecurity (FI) is defined as insufficient access to nutritionally adequate and culturally appropriate food to maintain an active and healthy lifestyle [1]. In 2017, 12% of U.S. households were estimated to experience FI [1]. A recent literature review estimates that U.S. college (i.e., post-secondary or higher education institution) students are at an elevated risk when compared to the general population, with 41% experiencing FI [2]. This has become a nationally recognized issue [3], as some college students experiencing FI have lower academic success as well as diminished health and well-being [4].

There are four components of U.S. household FI, originally defined by Radimer et al., [5]: 1) quantity; 2) quality; 3) psychological acceptability, and 4) social acceptability. The first component, quantity, indicates that households experiencing FI often restrict the volume and calories of their food. Quality, the second component, illustrates that food safety, nutritional density, and variety is also compromised. Individuals experiencing FI also report internal responses, such as anxiety or lack of control, and this is captured in the third component of psychological acceptability. The final component of social acceptability relates to eating patterns and acquisition strategies that many households experiencing FI may adopt due to insufficient resources. These expected components of FI, with the exception of social acceptability, were incorporated into the U.S. Department of Agriculture’s (USDA) questionnaires used to assess FI nationally [6]. Alaimo [7] provides a complementary conceptual model on household FI risk factors (e.g., employment, housing) which lead to specific experiences (e.g., worry about food, unsuitable food) to which coping mechanisms are applied (e.g., social support, self-reliance) leading to either amelioration of FI or contribution to individual-level consequences (e.g., hunger, low diet quality, psychological suffering). Though not specific to students, this model provides a more in-depth perspective that outlines how FI at the household level presents itself and produces negative physical and psychological consequences, if not alleviated.

Students may be at higher risk of experiencing FI due to the modern experience and population of post-secondary students in the U.S. College enrolment costs are expensive and have continued to increase over time [8]. The student post-secondary population has also evolved over time, with greater inclusion of historically underserved, minority and low-income students [9]. As universities have become more inclusive of diverse student demographics, some schools and faculty have developed new resources, policies, and support systems [10, 11], but their capacity to alleviate or prevent FI has not been clearly established [3]. In addition to these circumstances of higher education, many post-secondary students are transitioning into adulthood while enrolled [12]. The limited levels of prior experiences with food provisioning and resource management among many emerging adults enrolled in college may be associated with elevated risks of FI [13].

There is a second line of evidence that suggests high FI rates among students may be due to underlying issues and shortcomings of current surveying methodology. Calls have been made for general surveying protocol improvements [14]. More recently, investigations of college FI have evaluated the surveying protocols and identified potential issues. Though prior evidence supported the inter-changeability of the various forms of the Food Security Survey Modules (FSSMs), a scoping review of the literature found that the various forms of the survey produced variable estimates of FI among college students [2]. In addition, a psychometric evaluation of the FSSMs in a cross-sectional population of 18- to 24-year-old undergraduate students found that responses within the FSSMs did not follow the expected pattern of agreement [15]. Furthermore, the estimated prevalence of FI within a single sample was dramatically lower when screening protocols were used [15]. The FSSMs were developed in the late 1980s and early 1990s based on qualitative work where low-income mothers served as key informants [16]. Additional validation work was conducted for the general population [17] and other sub-populations [18, 19], but there has been a paucity of psychometric evaluations of the FSSMs for college students.

The initial population of mothers used to develop the tool likely differs from traditionally aged college students in a number of ways. In contrast to mothers, college students have varying levels of independence and are often not responsible for managing others in a larger household. Among students at 4-year universities in the U.S., only 6% are parents themselves [20]. In addition, it is common for young adults to report having limited experience managing food provisioning tasks [13]. Finally, the “income” of students is more difficult to define given the variability in types, formality, and distribution of financial support sources that students have [13, 15].

In addition to these differences between mothers and students, there is also the potential for differences arising from generational factors from when the surveys were developed. In the last three decades, the food landscape and provisioning tactics have changed, with more individuals eating foods prepared away from home and eating as a secondary activity while doing something else [21–23]. In addition, a recent investigation of 18- to 24-year-old adults found that taste, which is frequently the most important factor in food decisions, was closely followed by convenience and nutrition/health [24].

Based on the possibility that the FSSMs are incorrectly assessing FI among students, the current study was undertaken. The study aims to understand if fundamental interpretations of questionnaire items may explain, in part, high rates seen in the field. The two primary research questions under investigation are: 1) How do students interpret the USDA FSSM questionnaire items, and 2) How do responses of students experiencing FI compare with the theorized experiences and coping responses?

## Methods

### Participant recruitment

A random sample of undergraduate students between 18 to 24 years of age was recruited for an initial quantitative study [15]. To be eligible, respondents had to be undergraduate students, aged 18 to 24 years, and self-identify as fluent in English. Of the individuals who agreed to be contacted for future studies (*n* = 343), participants were categorized based on their FI status dictated by responses to an online 10-item USDA FSSM (items shown in Table 1). If individuals responded affirmatively to three or more items, they were considered food insecure; otherwise they were categorized as food secure. Thirty-two percent of participants experienced FI (*n* = 113) and the remaining (*n* = 230) were food secure. Among these two classifications, participants were randomly selected (using the *rand =* function in Excel [Microsoft, Redmond, WA, USA]) to be invited to participate, with efforts to recruit equal numbers of insecure and secure participants. When prospective participants declined to participate or did not respond after three invitations, they were replaced with a re-randomized selection from the remaining list of students.

**Table 1:** Questionnaire items and coding of response options as insecure or secure in the 10-item Food Security Survey Module^a^

Item	Affirmative (Insecure) Response(s)	Negative (Secure) Response(s)
10-item Food Security Survey Module:		
HH2. I worried whether my food would run out before I got money to buy more.	Often true, Sometimes true	Never true, Don’t know
HH3. The food that I bought just didn’t last, and I didn’t have enough money to get more.	Often true, Sometimes true	Never true, Don’t know
HH4. I couldn’t afford to eat balanced meals.	Often true, Sometimes true	Never true, Don’t know
AD1. In the last 30 days, did you ever cut the size of your meals or skip meals because there wasn’t enough money for food?	Yes	No, Don’t know
AD1a. In the last 30 days, how many days did this happen?	≥3 days	1–2 days
AD2. In the last 30 days, did you ever eat less than you felt you should because there wasn’t enough money for food?	Yes	No, Don’t know
AD3. In the last 30 days, were you ever hungry but didn’t eat because there wasn’t enough money for food?	Yes	No, Don’t know
AD4. In the last 30 days, did you lose weight because there wasn’t enough money for food?	Yes	No, Don’t know
AD5. In the last 30 days, did you ever not eat for a whole day because there wasn’t enough money for food?	Yes	No, Don’t know
AD5a. In the last 30 days, how many days did this happen?	≥3 days	1–2 days

Source: Bickel, G., Nord, M., Price, C., Hamilton, W., & Cook, J. (2000). Guide to measuring household food security. Retrieved from https://www.fns.usda.gov/guide-measuring-household-food-security-revised

### Data collection protocols

Students who participated were scheduled for a 60-min session where they completed paper-and-pencil questionnaires (both the 10-item USDA FSSM and demographic items) and were cognitively interviewed about the FSSM items. All items in the FSSM, including how they are coded as secure or insecure, are shown in Table 1. The survey they completed was stored, and a blank copy was used for their reference during the interview. The interview moderator read each FSSM item aloud and asked the participant to respond using think-aloud (i.e., “how did you go about responding to this question?”), comprehension (i.e., “what does the term X mean to you?”), emotional (i.e., “how did you feel about answering this question?”), and ease/confidence (i.e., “how sure of your answer are you?”) probes [25]. Additional open-ended probes (i.e., “can you tell me more about that?”) were used to elicit detailed descriptions of participants’ decision-making processes and the context that informed their choices. The interview moderator took notes during the session for later reference in creating codes. When the interview was completed, participants were compensated with $20 USD.

Though originally randomized to recruit equal numbers of 20 secure and 20 insecure participants, saturation was reached within the first eight interviews with food secure students. Saturation was evaluated by comparing the responses of food secure students with the primary research question (“How do students interpret the USDA FSSM questionnaire items?”) and noting the redundancy of responses. It was clear that the students categorized as food secure had no concerns about the adequacy of their financial circumstances and answered each FSSM item with this formulaic approach, so the investigators were confident that no new information would be revealed by additional interviews. Therefore, to maximize information gained, investigators decided that all future interviews scheduled should be with students experiencing FI. An additional three interviews were conducted with food secure students (for a total of *n* = 11 food secure interviewees) due to previously scheduled and confirmed sessions.

Interviews were recorded with a digital recorder and recordings were transcribed verbatim using an external transcribing service. Transcriptions were verified alongside the original recording by two research assistants, independently, to confirm accuracy. All participants consented in writing to participate, and the above protocols were approved by the institutional review board for research involving human subjects at the University of Illinois at Urbana-Champaign (IRB#18224).

### Analyses

Quantitative FI and demographic information were analysed and summarized using STATA/MP 14.1 (StataCorp, LP, College Station, Texas, U.S.). Transcripts were analysed and coded through a collaborative process by two researchers using a basic interpretative approach. The first phase of coding began with the generation of many codes (i.e., “open coding”) by each researcher independently, as new concepts occurred. Half of the interviews (*n* = 17) were randomly selected to be open-coded. Each openly coded interview was discussed in-person by both researchers to share newly identified codes and develop working definitions for identified concepts. A working codebook was developed based on these meetings and continually updated as new codes emerged or definitions were refined. In the second phase of coding, both researchers reviewed the codebook in light of the primary research questions before combining, refining, and categorizing codes. Formal definitions and examples were added to this draft of the codebook. In the second phase, selective coding was employed. In this phase, the codebook was used to “double-code” four transcripts, wherein each researcher independently used the codebook to code each interview using the predefined codes and marking transcript quotes that were not clear (i.e., statements that did not clearly fit into categories in the codebook). Agreement of researchers on these double-coded transcripts was greater than 80%. In the third, and final, phase the entire sample of transcripts was selectively coded by one of the two researchers, independently, using the final codebook and meeting to decide how to code any unclear quotes. The final transcripts with coded segments were entered into MAXQDA 2018 (VERBI GmbH, Berlin, Germany) to assist with final analysis and summarization.

## Results

### Participant characteristics

Sociodemographic characteristics of participants are shown in Table 2. Participants on average were 19.5 years old (± 1.2 years) and the majority (58%) were in their freshman or sophomore (i.e., first or second) year. A high proportion of respondents were white (58%), identified as female (70%), and were born in the U.S. (91%). Characteristics of participants deviate from the overall university’s undergraduate student body. The sample included more students who identify as white (58% vs. 45%) and Asian (21% vs. 18%) as well as more students who identify as female (70% vs. 45%) and less senior (final year) students (15% vs. 30%) when compared to the larger undergraduate student body. Living situation and financial resources available were diverse, with approximately half of participants using a dining hall meal plan (i.e., a university-based food service operation that is available to all students, faculty, and staff) and equal proportions of participants living in on-campus and off-campus residences. This high rate of on-campus living is likely related to the high proportion of first-year students in the sample and the university mandate that first-year students live on-campus and purchase a dining hall meal plan. On-campus living facilities at the university are in residence halls that allow students to have a miniature fridge in their room for food storage as well as access to shared cooking facilities (though the sufficiency and practical use of these facilities has been noted by previous students [13]). In contrast, off-campus living arrangements can vary in their capacity to support food storage and preparation but typically relies on more self-provisioning of storage and preparation equipment. Though participants estimated a variety of parental income levels, the majority (79%) perceived that they grew up in a middle-class household. The majority (79%) also received some financial support from their families.

**Table 2:** Sociodemographic characteristics of undergraduate students who participated in cognitive interviews and comparison with university’s undergraduate student body

Characteristic	All Participants ^a^ (*n* = 33)	Undergraduate Student Body ^a,b,c^ (*n* = 33,624)
Age (years), mean ± SD	19.5 ± 1.2	20.5 ± NR
College Classification, % (n)		
Freshman	30.3% (10)	20.3% (6837)
Sophomore	27.3% (9)	22.9% (7701)
Junior	27.3% (9)	24.7% (8287)
Senior	15.2% (5)	29.9% (10051)
Race/Ethnicity, % (n)		
White	57.6% (19)	44.8% (15061)
Black/African American	9.1% (3)	5.9% (1973)
Hispanic or Latino/a	9.1% (3)	11.2% (3748)
Asian/Pacific Islander	21.2% (7)	18.0% (6053)
Other/Mixed	3.0% (1)	20.2% (6789)
Gender, % (n)		
Male	30.3% (10)	54.6% (18345)
Female	69.7% (23)	45.4% (15267)
Has Dining Meal Plan, % (n)	51.5% (17)	NR
Residence Type, % (n)		NR
Greek (Fraternity or Sorority) housing	6.1% (2)	
Co-operative or communal housing	3.0% (1)	
Campus residence hall	48.5% (16)	
Off-campus apartment or house	42.4% (14)	
Living Situation, % (n)		NR
Lives alone	6.1% (2)	
Lives with other(s)	93.9% (31)	
Birth Country, % (n)		
United States	90.9% (30)	83.4% (28028)
Other country	9.1% (3)	16.6% (5569)
First-Generation Student, % (n)	24.2% (8)	20.0% (NR)
Sources of Financial Support, % (n) ^d^		NR
Family	78.8% (26)	
Employment	54.6% (18)	
Government	48.5% (16)	
Scholarship	54.6% (18)	
Loans	48.5% (16)	
Other	3.0% (1)	
Estimated Parental Income, % (n)		NR
Under $15,000	3.0% (1)	
$15,000 to $34,999	9.1% (3)	
$35,000 to $54,999	9.1% (3)	
$55,000 to $74,999	15.2% (5)	
$75,000 to $99,999	21.2% (7)	
$100,000 to $149,999	12.1% (4)	
$150,000 or more	18.2% (6)	
Don’t Know	12.1% (4)	
Perceived Familial Social Class, % (n)		NR
Lower class	9.1% (3)	
Middle class	78.8% (26)	
Upper class	12.1% (4)	
Familial NSLP use, % (n)	27.3% (9)	NR
Familial SNAP use, % (n)	3.0% (1)	NR

*NR* Not Reported, *NSLP* National School Lunch Program, *SNAP* Supplemental Nutrition Assistance Program; ^a^ Sum of column may not add to 100% due to rounding; ^b^ Division of Management Information publicly available student enrolment data; ^c^ Missing data from Division of Management Information: college classification (*n* = 748), gender identity (*n* = 12), and birth country (*n* = 27); ^d^ Sum of column will be greater than 100% as participants could select more than one source

**Fig. 1:**
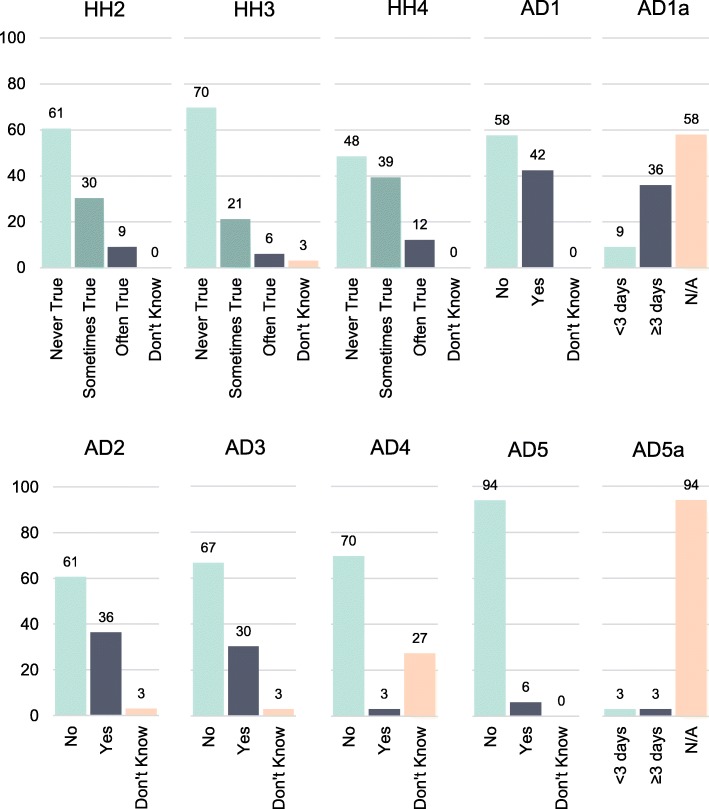
Prevalence of responses to each item of the USDA’s 10-item Adult Food Security Survey Module. Note. For exact wording of each Food Security Survey Module, refer to Table 1

Responses to each item of the FSSM are shown in Fig. 1 to illustrate the quantitative response pattern among included students. Theoretically, the items are ordered from lowest to highest severity, and the number of affirmative responses should decrease for each item based on its order in the FSSM. However, in the current sample the third item (affording balanced meals) and fourth item (cutting and skipping meals) were the two items most frequently affirmed. In addition, a considerable portion (27%) selected the “don’t know” option for item AD4, which asks about losing weight.

### Survey interpretation

Throughout the cognitive interview transcripts, students’ comments on their prior experience with the survey topics and their overall response to the questionnaire were coded to capture their general perspective on the survey. Responses were summarized and compared by resources available (e.g., dining hall meal plans, familial support) as well as FSSM classification.

When asked what they believed the survey measured, interviewees summarized the questionnaire in terms of hunger or general food behaviours among college students. Many used terms of “access,” “availability,” and “utilization,” which are some of the primary pillars used by the Food and Agriculture Organization (FAO) to assess population-level FI rates [26]. A minority of participants were familiar with FI and identified it in those terms.

Students comments indicated some general difficulties that arose with the questionnaire. Estimating the frequency of experiences over the last 30 days was perceived as difficult for many, so making reasoned guesses was common. For example, one student noted: *“For these last 30 days, I usually just think of the past two weeks cause I can’t really think further than that”* (18 years old, female, food secure). Both secure and insecure students mentioned generalizing over the semester period or referring to the previous week. For other students, the time range or qualifying monetary statements were completely overlooked. Though the interview took place immediately after participants completed the survey, it was common for students to forget individual questions or how they had responded. Students provided suggestions to modify the tool, particularly to include more response choices for the first three items to better capture their experiences.

Finally, a few respondents experiencing FI felt frustrated that their responses, which they thought were accurate, would categorize them as food insecure when they did not see themselves this way.

*“I think about food insecurity I don’t see myself as someone who is food insecure and so when I say yes to these questions, I’m thinking like I’m making myself sound like I’m food insecure when really I’m not”* (19 years old, female, food insecure).

These general-level interpretations of the survey provide insights on the way college students approach the FSSM, but greater detail was ascertained when the interviews were further analysed. The next section outlines students’ interpretations of key terms within the survey. Table 3 provides a summary of interpretational issues as well as participant quotes that illustrate each issue.

**Table 3:** Key interpretation issues on the 10-item Adult Food Security Survey Module in cognitive interviews with college students

Questionnaire item / phrase(s)	Interpretation issues	Example interview quote(s)
“Money for more” or another monetary clause in each item	- Monetary aspect of question overlooked by students - Variable interpretations based on heterogenous financial support sources (e.g., employment, savings, meal plan) - Dining hall meal plans used as sole reference and other support sources ignored	*“It’s not that it’s too expensive, no. It’s fine. I probably should have looked at it better. Yeah, I think I can afford all that with the foods I need.”* (21 years old, male, food insecure) *“I work for [a recreation centre]. It’s like an okay check. So, I literally call it my food money. I got that job for food, because it’s hard to force yourself to eat in the dining hall.”* (19 years old, female, food insecure) *“Like my meal plan and if I had any cash, like cash on me”* (19 years old, female, food insecure) *“I’m just going to think of the dining hall because that’s the, the easiest way to look at it. You know for [campus convenience stores] or uh, you know, somewhere I can buy food for myself, there’s a lot of different, you know, variables related to that, so we’ll just forget about that”* (18 years old, male, food secure)
“Balanced meals” in HH4	- Confusion between being able to afford healthy food and actual dietary patterns - Non-financial reasons for not eating “balanced meals” given	*“So I guess cost really wasn’t a driving factor for me starting to eat less balanced meals or anything. It just kind of happened.”* (20 years old, male, food insecure) *“I don’t want to go out to like buy all these ingredients. And it’s like such a hassle. So I think that’s why I can’t afford to eat balanced meals”* (18 years old, female, food insecure).
“Eat less than should” in AD2 and “cut size of meals” in AD1	- Items considered repetitive to students - Buffet-style of university dining halls made responses more complex	*“Um, yes because the last one was a yes and it was pretty easy since it was such a similar question. I felt like this question just reinstates the last question”* (18 years old, female, food insecure). *“I did eat less because I wasn’t eating the regular two meals* *per day. So one meal a day. So I would end up eating a lot more just cause I was afraid like ... I wasn’t sure of the next time I would be able to eat, which is why I tend to overate at the dining hall”* (18 years old, female, food insecure)
“Hunger but didn’t eat” in AD3	- Various interpretations of “real hunger” and whether experiences of students counted as hungry	*“Hungry is kind of like a broad definition you know, like I was not like starving, but I mean I’m hungry right now, and I skipped lunch. (Soft laugh) But, it’s not like painful hungry or like horrible hungry. It’s fine*” (18 years old, male, food insecure).
“Lost weight” in AD4	- Students did not monitor and were not aware of their weight	*“Uh, I think I put “no”. And I should have put “don’t know” cuz I don’t know if I’ve lost weight. I’ve only been to the doctor once at the beginning of the school year and I don’t have a scale.”* (19 years old, female, food insecure)

#### Money for more

The stipulation that experiences are related to monetary restraints is present in all FSSM items with clauses such as “money to get more” or “because there wasn’t enough money.” However, these clauses are not consistently interpreted due to the heterogeneity of student resources. Resources ranged in formality and stability as well as their basis as financial or specifically food-related (i.e., dining meal plans or physical food available). Resources could include, but were not limited to, parental (or other family) support, a dining hall meal plan (of which, there were four offered by the university), wages from prior or current employment, food provided by friends or roommate(s), food available at events or provided by organizations, and financial support provided through scholarships or other aid sources. Given the variety of resources available to students, the term “money to get more” was interpreted in various ways.

*“How much money’s in my bank account. All the money that I earned over the summer”* (19 years old, female, food secure).

*“I didn’t have to worry about [my food] necessarily running out and then paying another installment because I pay [for the dining hall] upfront at the beginning of the semester for the food”* (20 years old, female, food secure).

*“When I think of money for food, I’m thinking about um what I have for meal swipes and [campus convenience store] credits”* (19 years old, female, food insecure).

For those that had dining hall meal plans, they often used this as a sole reference and ignored other resources when answering items, serving as a cognitive heuristic to ease their response choice. Some interviewees suggested that meal plans be listed explicitly alongside references to money to make it clear that they should be referencing both resources.

When comparing students by FI status, students with food security were more likely to indicate parental support and/or a meal plan provided most of their food resources. No students with food security mentioned current employment, friends/roommates, or events/organizations as food resources. Students with food security were also less likely to mention worrying about their resources or managing them tightly. Students experiencing FI, in contrast, referenced a spectrum of food and financial resources. Despite this variety, and often piece-meal resource landscape, many students experiencing FI interpreted “money for more” as simply their meal plan, the money in their bank account, or a pre-determined food budget. A few students experiencing FI had trouble voicing what they were referencing when they saw the financial qualifier. One student experiencing FI described a working tabulation of their expenses and expected influxes of financial support as their reference point, revealing the chaotic nature of their financial situation.

#### Balanced meals

The third item of the FSSM, often referred to as HH4, asks whether participants can afford balanced meals. Interviewees were asked to define their interpretation of the term balanced meals. The most common definition, among both insecure and secure students, were meals where all (or most) food groups were present. Many referenced U.S. dietary guidelines – either the Food Pyramid or MyPlate – as their idea of a balanced meal. Other respondents had even greater standards that incorporated exclusively organic items or included supplements. One student experiencing FI felt assured they had a balanced diet because they analysed their nutritional intake. In contrast, most respondents felt their diets fell short of these standards. However, it was difficult for some participants to separate whether this was due to financial restraints. One student answered affirmatively because they felt that inherently their diet was unhealthy if they spent little on it: *“I just don’t like spending so much money on like super healthy things cause I feel like they’re really expensive”* (21 years old, female, food insecure). In contrast, students with food security who did not eat healthfully felt it was straightforward that this was a personal choice or influenced by other priorities: *“So... couldn’t afford to eat balanced meals. I can afford it. I would just have to spend more”* (19 years old, female, food secure).

Among students on meal plans, they felt they could make balanced meals in the dining hall but were restricted by the hours they could attend and number of meals on their plan. If students could not attend regular dining hours, they used meal plan credits at on-campus convenience stores but creating balanced meals was perceived as difficult. *“Like with [campus convenience store] credits, like the chips thing. You shouldn’t eat chips as a meal, but when you have nothing else to do, you eat just water and chips”* (19 years old, female, food insecure).

The balanced meal question elicited negative feelings among some respondents experiencing FI, as they reflected on how financial constraints impacted their diets: *“It made me a bit sad because I realized that I’m eating pretty terribly, and I should be supporting my body better than I am”* (18 years old, female, food insecure). Many students experiencing FI felt shame that they were not meeting perceived cultural standards to eat healthfully. However, a minority of students categorized as food insecure felt that poor dietary habits were the norm for students and not something to worry about: *“It seems like such a college kid problem. That’s why I laugh so much, cause it’s just ramen is just what you eat”* (20 years old, female, food insecure).

As interviewees discussed the item, participants categorized as food insecure who had not considered the financial restraints aspect of the item expressed a desire to change their response. They remarked that talking about it made them consider non-financial factors like class schedules and extracurricular activities that impacted their dietary choices.

#### Eating less than should

The sixth item of the FSSM asks whether a respondent ate less than they felt they should (item AD2). This item follows another item asking whether respondents skipped or cut the size of their meals (item AD1). Students were comfortable and confident responding to these items but indicated that they seemed similar. Interviewees felt that if they had answered “yes” to skipping meals then that meant they were eating less and so they should answer “yes” again.

A few students reported unique approaches to this item. One participant used their hunger levels to determine if they ate less than they should. Another student experiencing FI approached AD2 by evaluating their grocery shopping and describing how they spent less money on groceries than they actually ate because they relied on free food resources:

*“If I spent $40 a week or something like that on food and ... I’m trying to put this to number because that’s how my brain sort of works, but I eat 45-ish dollars worth of food over that week, that five dollars that came from eating something at some kind of organization, that’s sorta how I answered it”* (21 years old, male, food insecure).

For students on meal plans, their responses were complicated by the buffet-style used in the facilities. They were inclined to answer negatively about eating less because they could eat as much as they would like during a meal, even if they only ate one or two meals a day.

Despite these remarks about the similarities of AD1 and AD2, participants did not respond to them identically when they filled out the survey (see Fig. 1). Of the 14 affirmative response to AD1, only 10 respondents also affirmed AD2. In addition, there were two respondents who affirmed AD2 after responding to AD1 negatively. Therefore, participants had some variation in interpretation and actual survey responses that were not widely reflected in the cognitive interviews. This may, in part, be due to the lack of memory and concentration when completing the survey.

#### Real hunger

As interviewees explained their response rationale to the seventh item of the FSSM (AD3), which asks respondents whether they were hungry and did not eat, the term “real hunger” was commonly used. Students thought of stereotypical hungry people and not themselves: *“People usually associate are you always hungry with like homeless and the hungry, and you got to feed the hungry and the poor”* (18 years old, female, food insecure). Discomfort answering this item arose among some students experiencing FI when they had otherwise avoided thinking about their experiences of physical hunger. Comments among students categorized as food insecure revealed they compared their experiences of hunger against their personal standards of “real hunger,” often discounting their situation.

*“Like 24 hours is like hungry. Like I don’t know. I feel like if I don’t wait that long then I’m not really, then it’s like, ‘you’re just a little bit hungry,’ or something like that”* (19 years old, female, food insecure).

The distinction of real hunger versus personal hunger was also mentioned by students categorized as food secure. However, students with food security could easily identify that their experiences of hunger were not related to money shortcomings in contrast to students experiencing FI who had to judge if their hunger was “real” enough.

#### Weight loss

Respondents are asked if they lost weight because they did not have enough money for food in the eighth question (AD4) of the FSSM. Many interviewees indicated that they did not know their weight because they were not interested in tracking it, did not own a scale, or did not know where they could weigh themselves on campus. However, for those that knew their weight, the quantifiable aspect of weight loss made this item straightforward to answer. Students who did not regularly weigh themselves felt the item was difficult unless they had opted to select “don’t know.” However, some students who selected “don’t know” would apologize because they believed that this was a “wrong” answer for surveys.

Some respondents experiencing FI answered negatively to this item but revealed there were factors outside of food and finances that impacted their answers. For example, one respondent relied solely on their meal plan for food but had other commitments during open dining hall hours: *“I think I’ve lost some weight, but I don’t know. If it is, it’s not because of lack of money for food, it’s because of lack of time for food”* (19 years old, female, food insecure). Other students experiencing FI felt that no matter how much they ate, their weight would not reflect it because they had elevated metabolism levels or were still growing. One student categorized as food insecure who did not weigh themselves tried to estimate if they lost weight based on their energy level but found it difficult to understand if their fatigue was a product of malnutrition from skipping meals or from academic pressures. Another student experiencing FI felt their weight fluctuated week to week based on how tightly they were rationing their food, but they chose to answer “no” because short-term weight changes were not concerning to them.

Respondents with food security were comfortable answering this weight-related item. When asked if the item elicited any emotional response, students talked about how dissatisfaction with one’s weight would make someone uncomfortable answering this question. This idea that high weight was negative and the item may provoke shameful feelings, as well as the converse that low or losing weight was inherently positive, was often discussed by both secure and insecure students.

### Food insecurity experiences

Interviews with students categorized as food insecure were used to further understand the process and circumstances of student FI. Transcripts were compared with the four components of household (or individual) FI, originally conceptualized by Radimer et al. [5], which were used to develop the USDA FSSMs [6]. These components are: 1) quantity; 2) quality; 3) psychological acceptability, and 4) social acceptability.

#### Quantity

The first component of FI is quantity of food – specifically that individuals have access to sufficient calories. It was common for students experiencing FI to decrease their intake by cutting meal size or skipping meals. A few students mentioned being hungry or not eating to fullness, but these instances were often disregarded and not considered “real hunger.” Whether these experiences translated to suboptimal caloric intake was unclear as some felt they had adjusted to the new eating patterns: *“I guess because of that worry that I won’t have enough credits, I started eating less and it just became less of a worry, became a new schedule, new habit”* (19 years old, female, food insecure). However, there were a few students who explicitly indicated making severe caloric restrictions. One student estimated they would restrict their intake to 400–600 kcals per day. Another student who went entire days without eating avoided expending energy (i.e., not bicycling to campus) on these days.

For students with dining hall meal plans, skipping meals did not always translate into lower overall intake because of the buffet-style service at the meals they attended.

*“I feel like to say that I don’t have enough money for food is like a bit of exaggeration cuz with the meal plan like you have enough, it’s just at weird intervals”* (19 years old, female, food insecure).

#### Quality

Individuals experiencing FI may compromise the quality and safety of their foods, elevating their risk of nutrition inadequacies or illnesses. Most students categorized as food insecure reported eating low quality food, referencing low nutritional value or cost. One FI student stated *“I’m usually eating like dollar cheeseburgers from [fast food restaurants], or $0.50 cans of [prepared pasta], or just shit food”* (20 years old, female, food insecure). Students would forgo purchasing healthy items to save money, instead eating foods that satiated them: *“As much as I want to get vegetables, vegetables are not going to fill me up”* (21 years old, female, food insecure). The exception was among students with meal plans who could eat a variety of healthful foods in the dining halls, given the buffet-style distribution. Though, a few students categorized as food insecure were concerned about the taste quality and food safety standards in these facilities.

Cost and financial restraints were a dominant factor in food decisions among those experiencing FI, but many students also referenced the role of convenience and other priorities in diminishing the quality of their diets. One student reflected that: *“It’s just like it’s so much easier to buy cheaper, non-healthy foods on campus than it is to buy healthy foods”* (22 years old, female, food insecure). Limited preparation capabilities or skills were often cited as rationale for why students purchased and ate foods they perceived as lower quality. Many, though not all, students reported their diets were low quality, but it was unclear whether they would be at risk for nutritional inadequacies. It is also unclear whether these patterns vary from the average student with food security who sees food as an area where costs can be reduced. The reference for “quality” is ambiguous for most respondents, created based on personal and cultural norms. However, one interviewee experiencing FI used their studies in biochemistry to evaluate the nutritional adequacy of their diet.

#### Psychological acceptability

The third component captures how an individual experiencing FI responds internally to their situation. Responses often included anxiety about food and feelings that one’s choices are unacceptably restricted or out of their control. For interviewees experiencing FI, unplanned expenses felt alarming and like tests of adulthood. When students felt they needed support from their family, they often reported hesitating to reach out and feeling like a burden.

Among students on dining hall meal plans, many reported feeling that they were restricted to eating only what the plan could cover: *“Like I can’t eat this meal because I’m gonna like- because I’m not gonna- I’m gonna run out of [meal credits]”* (19 years old, female, food insecure). This perceived restriction was common even if they had resources to cover external expenses. One student who had external funds noted that: *“I just hate spending my money on food”* (18 years old, female, food insecure). Trying to restrict one’s self only to the meal plan allotment produced a semi-daily task of checking their plan’s remaining balance online or conducting “mental math”: *“How long can I last without um using a credit? Can I sacrifice a credit for another day*?*”* (19 years old, female, food insecure). This task often produced anxiety: *“You don’t even get enough for two meals a day, so I was- I do worry about that a lot*” (19 years old, female, food insecure).

Many students felt powerless about their situation: *“There’s nothing wrong with skipping meals if you have to do it. You do what you got to do”* (21 years old, male, food insecure). Many students rationalized their restrictions as normal, regardless of the severity they described. Feelings of powerlessness were more common among students who discussed food access issues before enrolling at the university, as the circumstances were seen as normal to them and they had not expected things to be different in college.

There were also responses indicating that the restrictions students made were not worrisome or they felt not consciously impacted by them. After indicating they skipped meals or were eating unbalanced diets, students would say they were not concerned about these practices.

*“When you’re at a dining hall, it’s like so many options and opportunities so even though you have a strict amount of time you can go, that’s not, you still have access to food … I would consider it a moderate inconvenience*” (19 years old, female, food insecure).

#### Social acceptability

The final component, social acceptability, evaluates whether an individual has to deviate from normal food acquisition and consumption patterns due to restriction. Specifically, individuals experiencing FI may eat an abnormal meal pattern or they may acquire food through non-conventional sources. Commonly, a normal meal pattern is perceived as three meals a day with flexibility to accommodate additional snacks. However, individuals across the U.S. have dietary patterns that vary in number and size of eating occasions, regardless of their financial resources.

It was common for students categorized as food insecure to report eating two meals or less a day and supplementing this with smaller snack items. Many referenced using inexpensive low-quality snack items to substitute for full meals. However, it was not always clear if students chose their eating patterns or were “forced” into them through external circumstances. When a student was reflecting on whether they would prefer not to skip meals and eat three meals a day, it was difficult for them to consider because they felt that was an unachievable scenario and skipping meals was described as *“So normal to me now”* (19 years old, female, food insecure).

One student provided insights on how financial restraints broadly impacted her eating: *“Always trying to be mindful of money kind of has made me like a little less inclined to just feed myself naturally like how I would need to eat”* (19 years old, female, food insecure). Though specific eating patterns and choices made by students experiencing FI due to limited finances were not frequently described as socially undesirable, a few specific instances clearly deviated from expected practices. For example, restricting one’s self to eating a single canned food for dinner: *“I will eat one can of beans and then I’ll say okay I can’t eat anymore because I need this next can of beans for tomorrow”* (19 years old, female, food insecure). Or, drinking water instead of eating meals and avoiding food stimuli to ignore one’s hunger: *“I’ll drink water, and I just don’t walk past like [fast food restaurant], or something like that, or like where I can smell it”* (19 years old, female, food insecure). In addition, a few students with meal plans would discuss needing to binge eat in the dining hall because of the infrequent eating occasions they had available to them with plans that covered two or less meals per day.

There was also the potential for social consequences based on the food management processes students had to undertake. One student participated in social gatherings, but would lie about why they were not eating: *“I just, I say I’m not hungry or that kind of stuff, like I come up with excuses”* (21 years old, female, food insecure). One student received additional food money from their parents to eat with friends: *“Cause my parents also didn’t want me to like back out of like social events just cause I was out of money”* (18 years old, female, food insecure). Other students described declining offers to eat with friends because of their financial restrictions. A few students mentioned using less conventional food resources (i.e., food pantries, free food events, community organizations) to supplement their diets, but this was not common for most students categorized as food insecure.

### Food insecurity coping strategies

The final analysis of food insecure interviews evaluated whether students enacted theorized coping mechanisms. Alaimo [7] outlined how individuals responded to and coped with FI based on several seminal works. Coping responses are broadly categorized as self-reliance, seeking social support, seeking formal support, and seeking emergency support.

#### Self-reliance

Relying on one’s self (e.g., resource management, rationing) and hiding their personal situation from others is one coping mechanism. Rationing food resources was common for many students experiencing FI. This included portioning out food items: *“For breakfast or something, I’ll only eat like half of it. And then that makes it last twice as long, instead of eating the whole bagel”* (19 years old, female, food insecure) and rationing dining hall meal credits.

As students experiencing FI became accustomed to being at the university, many reported learning and adapting to operate within their restraints. For many students, this was their first time being responsible for managing their money and food resources. One student described that: *“It was kind of different because I was used to somebody having meals for me, now I have to figure out, ‘Okay, can I eat now? Or can I eat later?*’*”* (19 years old, female, food insecure). Learned approaches to self-reliance included building cooking confidence, meal prepping, identifying free food resources, learning about the dining hall hours and meal structure, budgeting, or making various adjustments to their eating pattern.

Many interviewees discussed feeling like a burden if they relied on others. For example, one student remarked that: *“I would hate to kind of be the inconveniencing factors in someone’s life, even if it’s like $10 or $20. It’s $10 or $20 they could have spent elsewhere”* (19 years old, female, food insecure). Many students were acutely aware of the financial support that their families provided to pay for other expenses:

*“I don’t really want to bother them with like paying for more than what they’re already paying for because like the meal plans are like really expensive, and like I don’t- I don’t know, I guess I feel uncomfortable asking them for more money”* (19 years old, female, food insecure).

Additionally, some students felt alienated and that they should hide their situation from others. One student avoided discussing their situation with others: *“If I ever told someone that I’m spending like 10 dollars on food a week they would be pretty shocked and they’d think that I’m not eating properly”* (19 years old, female, food insecure). Another student reflected that it would be shameful to discuss financial issues with other students.

*“When you’re on campus, there’s like a healthy amount of people that are really like wealthy. Like I’ve even experienced that. And it’s like you don’t want to have like a conversation with somebody who’s like, can get like whatever they want while you can’t afford something”* (19 years old, female, food insecure).

When students experiencing FI discussed their approaches to food resource management, it was common for students to rationalize their situation and dismiss it as normal. One student felt: *“That’s not food insecurity, not eating breakfast (laughs), but you know a lot of people do that”* (19 years old, female, food insecure). The student who reported eating single food items for meals, such as a can of beans, asserted that: *“I am getting adequate nutrition and therefore it doesn’t matter whether or not I’m eating meals”* (19 years old, female, food insecure).

#### Seeking social support

Another coping response is to seek support from social networks (i.e., friends, family, and community members). It was commonplace for students experiencing FI to discuss various members of their social network that provided financial and food support to them. When friends were referenced, support was often informal and did not require students disclose any hardships.

*“My roommates usually make those oven pizzas and stuff and like there’s obviously more than just one person to eat, so ... and they don’t want to save it because it’s just going to get kind of weird in the fridge, so they always like give me a slice”* (21 years old, male, food insecure).

Informality and anonymity was also true when students sought support from community organizations or events. They were able to receive free food without indicating any atypical need by attending events or club meetings on campus.

*“There’s a lot of shame around food insecurity, I think it’s great to have events there, you know for cultural or you know some sort of gathering of sorts (laughs) that have food. Cuz I’d, I did that too. There’s like a lecture series on Friday that I like to go to”* (19 years old, female, food insecure).

However, when needs became greater than single items or meals, students often had more hesitations about reaching out to their social support systems. Oftentimes their desire for independence and “status as an adult” was threatened if they had to call their parents or other family members for money. For some, this meant they would skip meals instead of reaching out. For others, they acknowledged their desire for independence, but ultimately decided to ask for help: *“The fact is I could if I really don’t have anything to eat, like I’m never going to skip a full meal and just be hungry”* (19 years old, female, food insecure).

#### Seeking formal support

Seeking formal support to increase one’s food supply through government assistance or employment is another coping strategy. Many of the students experiencing FI were employed while enrolled in school or had worked to ensure they had savings accrued before the semester. One student, who had initially answered the FSSM as food insecure during the screening process, felt their circumstances were in the midst of change because they had attained a job between the initial survey and the interview process. As a vegetarian using the dining hall meal plan for the majority of their food, this student felt their options were limited. They reached out to dining services to request menu alterations but also noted: *“That also led me to apply for jobs”* (19 years old, female, food insecure), so they could buy food from external sources.

Though likely a large source of financial resources for food, no other students discussed their jobs explicitly as needed to purchase food. One student specifically sought employment to build her social network and indicated: *“Like the money is kind of like a bonus”* (19 years old, female, food insecure). Only one student reported using SNAP benefits, in addition to their other resources (e.g., employment, family).

#### Seeking emergency support

The final coping response is seeking support from emergency sources. This can include visiting food pantries, begging for food, and stealing food. Emergency support seeking was not common among the interviewed students experiencing FI. No students discussed stealing or begging for food, but food pantries were discussed. However, many times when food pantries were mentioned it was in the context of helping “real” hungry people such as individuals experiencing homelessness or families struggling to feed their children. Some noted that food pantries would be helpful to serve other students experiencing FI, but there were only two students interviewed who explicitly discussed personal food pantry use. They both lived off-campus and used an on-campus food pantry one time per week as a supplement to purchased groceries. One of the students using the pantry connected this to her cultural upbringing and explained her perspective: *“I was always taught when it comes to food you don’t guess it. You should never be starving”* (21 years old, female, food insecure). In contrast, when other students discussed food pantries, they felt they would be too prideful and uncomfortable to use them.

## Discussion

The objective of the current study was to capture how traditional undergraduate college students understand the 10 items of the FSSM that query about adult experiences with FI. In addition, interviews with students experiencing FI provided ample perspective to compare their experiences with theorized elements of FI. The interpretations of key aspects of the FSSM indicate the content validity for this population may be compromised. Of particular concern were the interpretations of “money for more” clauses, responses to the “balanced meal” item, and difficulties addressing questions about weight loss related to food insufficiency. In addition, students experiencing FI reported a variety of coping responses and experiences, indicating that the theoretical “managed process,” developed for the general population [5, 16], may not be appropriate for post-secondary students. It is critical that future research distinguish experiences related clearly to FI from separate experiences that are related to being an emerging adult or coping with enrolment as a college student.

The potential misinterpretations of many key terms in the FSSM aligns with prior studies that have indicated the current surveying methodology may be inappropriate for the college student population [2, 15]. Nikolaus et al. [15] found that item infit and outfit statistics were compromised for the “balanced meals” item as well as the items related to losing weight and skipping meals. This corroborates the various interpretations and response patterns found in the current sample of students. The high rates of FI reported in the recent scoping review [2] may, in part, be related to the various interpretations and response issues that students reported having related to the FSSM.

The qualitative findings that there may be inaccuracies in the FSSM among college students are similar, in some respects, to previous work using qualitative investigations to understand how other populations understand the FSSMs. Foster et al., [27] compared mothers’ and fathers’ interpretations of the 18-item USDA Household FSSM, finding that fathers had unique interpretations of the terms “balanced meals” and “household.” In addition, they also reported that fathers had limited anxiety as a response to FI [27]. Though gender was not a segmentation strategy in the current study, many students rationalized or normalized their experiences with FI. This suggests that psychological acceptability may be an inessential element assessed when evaluating college FI. Similar to the current findings, previous work found that the term “balanced meals” was also a potential source of error and misinterpretation when this was evaluated through interviews with Hawai’ian residents [19]. Though quality is an essential component of FI, it is unclear if using the key term of “balanced meals” is the best method. This may be particularly true among high-school educated young adults pursuing a college education. The findings illustrated the high standards with which many students compared their diets. Though nutritious dietary patterns are ideal for all Americans, in a commentary on measuring FI, the quality aspect is described as acquiring essential nutrients, not optimizing diet quality [14]. It is important that the indicator of quality in a FSSM evaluate potential compromises and undernutrition, not common difficulties that many adults face in meeting dietary guidelines [28]. In addition, an item assessing dietary quality needs to ensure that respondents distinguish the affordability of eating balanced meals from the action of eating balanced meals to remove the role of other influences on dietary patterns.

In addition to the investigations of specific FSSM items, the current results also provided an in-depth understanding of students’ FI experiences. This is not the first qualitative investigation of FI among college students [29, 30], but to the authors’ knowledge it is the first to formally analyse experiences against theoretical elements of FI. Common sentiments that students experiencing FI prioritize satiety, cut the size or skip meals, and eat lower quality convenience foods was also reported in focus groups of college students in California [30]. In later semi-structured interviews among a separate sample of students in California, the impact of FI on students’ psychological wellbeing, producing anxiety, and social relationships was described [29]. The current results indicate there is similarity between the experiences described by students enrolled in public California universities and students experiencing FI in the current study. However, the adequacy of the FSSMs to capture the California university students’ sentiments was not described.

Students in the present study discussed a myriad of factors that are not captured in the current FSSMs. Many students had dining hall meal plans, and the structure of this university-provided food influenced students’ eating behaviours and complicated interpretations of FSSM items. Students with meal plans would report restricting themselves to eating only the 10 or 12 meals per week that some plans would cover; self-described binge eating would take place to maximize these meals. Other students would discuss not using all meals on their plans, due to preferences and desire to eat elsewhere or due to difficulties in attending dining halls during their open hours. These qualitative findings may provide some underlying insights for the recent reports that FI is related to meal plan use and the commonality of unused meals on plans [31]. For students accessing food off-campus, time and transportation issues were discussed. Regarding eating balanced meals, students noted the multiple inconveniences that would be posed by navigating public transportation, transporting ingredients to their residence, and preparing the items as a meal. Other researchers have noted how the remote locations of some campuses can create food access issues [32], which may influence FI among students. Students in the current study attended a campus that has multiple food retailers in the vicinity, with one grocery store located half a mile from the student union building. However, the role of food provisioning skills and time constraints of undergraduate studies may influence how students perceive their larger food environment.

This study, alongside other quantitative investigations [2, 15], reveals current methods may not be accurately estimating the FI among students. Future research studies will need to, first, evaluate and confirm if these findings are similar or different when surveying different student populations. Specifically, qualitative and quantitative psychometrics tests are warranted in students at 2-year schools, non-traditional aged students, and among graduate students. Beyond these replications, in-depth investigations developing and testing a new FI questionnaire for this population may be valuable. However, drastic changes may complicate comparisons with national FI estimates. It is possible that making subtle modifications or adding supplementary items to the existing FSSMs could be suitable to address psychometric issues. Though speculative, some of these minor questionnaire modifications could include making the financial clause more salient by moving it to the beginning of each item, listing meal plans or other common financial resources alongside money, and providing more than 3 response options. Alternatively, many studies have investigated very low food security, the most severe form of FI, among students [33], and it is possible that this specific classification could better distinguish FI students. Tests of concurrent validity, evaluating FI alongside other theorized risk factors (race, financial support, etc.), consequences (diet quality, mental health, etc.), and proxies for FI, would also be a valuable contribution to the literature. Until appropriate measures of FI among students are established, evaluations of suggested initiatives [3] will be compromised.

The findings of the current study should be made with considerations to the limitations of the study design. The interviews were limited to the 10 adult items in USDA FSSM. Recent psychometric research indicates that the most accurate assessments of FI may be produced with the addition of a 2-item food sufficiency screener [15], so qualitative investigations that include these additional questions may be valuable. In addition, the qualitative approach naturally limits the ability to quantify the impact of the issues identified. Given the labour-intensive needs to conduct in-depth interviews, the number of students included in most qualitative investigations, including the current study, was limited. However, saturation (i.e., the continued vocalization of common topics and themes) was achieved within the included interviews for the primary research question related to the appropriateness of the FSSM. The study was limited to one 4-year university with traditionally aged undergraduate students. Students in this sample were enrolled at a public university in a small city that enrols approximately 50,000 students with “Very High Research” activity and is considered “more selective” based on the standardized test scores of the admitted first-year students [34]. During the year that the study was conducted, the annual cost of attendance for undergraduates at the university ranged from $30,000 to $52,000 [35]. Therefore, generalizability to other contexts is limited.

## Conclusions

It is clear, based on the current results and other published literature on the topic, that FI is a pressing issue among some post-secondary students in the U.S. However, the current FSSMs may not be the best tools to assess FI in this population. Testing modifications to the current FSSMs or development of new tools will be essential to better prevent and alleviate these issues among students. Diverging opinions have been debated with some individuals asking whether students experiencing FI should be enrolled in college, a large personal expense. This sentiment was reflected by one interviewed FI student.

*“There’s so many- So many angry older people in line who would like to remind you that you don’t have to go to college, or you don’t have to drive a car, or you don’t have to- You don’t have to have nice clothes. If I was really hungry, I shouldn’t have a cell phone. If I was really hungry, I wouldn’t have like a laptop you know”* (20 years old, female, food insecure).

However, in the current economy, a post-secondary education in the U.S. is no longer considered optional by many based on the long-term implications of a college education [36]. Given this, it is likely that the proportion of young adults seeking college degrees will only continue to grow. To ensure that FI is prevented, alongside the myriad of downstream health and social consequences, accurate surveys to measure the current issues and changes over time will be essential.

## Data Availability

The datasets generated and/or analysed during the current study are not publicly available due to protect individual privacy, but de-identified portions of the data are available from the corresponding author on reasonable request.

## Abbreviations

FI: Food insecurity
FSSM: Food Security Survey Module
USDA: U.S. Department of Agriculture
